# SREBP1-SCD1 enhanced MUFAs Biosynthesis drives Nutrient Deprived Pancreatic cancer cell Ferroptosis Resistance

**DOI:** 10.7150/jca.114356

**Published:** 2025-09-08

**Authors:** Zhengyang Zhang, Xiaojie Cai, Yi Gong, Aihua Gong, Xiang Liao, Jie Gao, Dongqing Wang

**Affiliations:** 1 Institute of Medical Imaging and Artificial Intelligence, Jiangsu University, Zhenjiang, 212001, China.; 2 School of Medicine, Jiangsu University, Zhenjiang, 212013, China.; 3 Department of Radiology, Changshu Hospital Affiliated to Suzhou University, Changshu, 215500, China.

**Keywords:** Ferroptosis, nutrient deprivation, MUFAs, rapamycin, pancreatic cancer.

## Abstract

**Background:** As the most disastrous tumor microenvironment of pancreatic cancer, nutrient deprivation determined various cancer cell biology, especially the cell death resistance. Our objective is to elucidate the role of nutrient deprivation in ferroptosis resistance of pancreatic cancer cells and to explore potential therapeutic strategies to overcome it.

**Methods:** To replicate the nutrient-deprived tumor microenvironment, pancreatic cancer cell lines (PANC1 and Patu8988T) were cultured in Dulbecco's Modified Eagle Medium (DMEM) supplemented with 2% fetal bovine serum (FBS). Ferroptosis was assessed by Cell Counting Kit-8 (CCK8), Malondialdehyde (MDA) assay, and C11 BODIPY staining. The signaling activity was assessed via western blot and quantitative reverse transcription polymerase chain reaction (qRT-PCR), respectively.

**Results:** Ferroptosis inducers promoted pancreatic cancer cell death could be significantly reversed under nutrient deprivation condition. Nutrient deprivation upregulated the expression of SREBP1 and SCD1, leading to increased intracellular levels of monounsaturated fatty acids (MUFAs). Genetic knockdown of SREBP1 or SCD1, as well as treatment with rapamycin (an mTOR inhibitor), reversed the nutrient deprivation induced increase in mature SREBP1 and SCD1 expression and restored lipid peroxidation both *in vitro* and *in vivo*. The synergistic application of sorafenib and rapamycin yielded a profoundly efficacious therapeutic outcome *in vivo*.

**Conclusion:** Our findings demonstrate that nutrient-deprived pancreatic cancer cells adaptively enhance MUFA biosynthesis through the SREBP1-SCD1 axis, conferring resistance to ferroptosis. This resistance can be effectively overcome by combination therapy with sorafenib and rapamycin, offering a promising strategy to target the ferroptosis vulnerability shaped by the pancreatic tumor microenvironment.

## Introduction

Due to the intrinsic therapy resistance ability, pancreatic cancer is a leading cause of cancer-related mortality globally with the five-year survival rate remains below 12%^1^. Pancreatic cancer therapy resistance is ascribed to both cancer cell death resistance and the complexities of its tumor microenvironment^2^-^4^. However, the exact mechanisms by which the tumor microenvironment determines the cancer cell death resistance is not completely understood and deserves deeper investigation.

Ferroptosis, a novel form of non-apoptotic cell death characterized by iron-dependent lipid peroxidation, has been implicated as a robust modality in refractory cancer treatment^5^,^6^. The combination of gemcitabine and sulfasalazine exhibits a synergistic effect in inducing pancreatic cancer ferroptosis 7,8. Unlike other forms of programmed cell death, the execution of ferroptosis is mediated by lipid peroxidation and membrane damage^9^,^10^.Thus, fatty acid metabolism and lipid species, such as monounsaturated fatty acids (MUFAs) and polyunsaturated fatty acids (PUFAs)-containing phospholipids, determined the ferroptosis sensitivity^11^. It has been reported that the relative content of polyunsaturated fatty acids (PUFAs) and monounsaturated fatty acids (MUFAs) dictates the execution of ferroptosis^11^,^12^. Sterol regulatory element-binding protein 1 (SREBP1), a crucial transcription factor that regulates de novo lipid biosynthesis, is also involved in the ferroptosis sensitivity 13,14. In addition to the cell intrinsic metabolism, tumor microenvironment could regulate cancer cell ferroptosis sensitivity in a direct or indirect manner. For example, a hypoxic tumor micro-environment can mediate tumor cell ferroptosis resistance by activating HIF1α and upregulating various anti-ferroptosis molecules such as SLC7A11, GPX4, TFRC and FTH1^15^-^17^.

Due to the imbalance of blood supply and rapid proliferation of the cancer cell, dysfunction of vessels, nutrient deprivation is one of the most characteristic features of the pancreatic cancer microenvironment 18. Nutrient deprivation is notorious for its effect on cancer cells progression. White et al. found that serum deprivation culture conditions can mediate prostate cancer cells' adaptation to and survival under oxidative stress^19^. Additionally, nutrient deprivation conditions activate the autophagy pathway in pancreatic cancer cells, not only promoting their survival but also enhancing stem cell activity^20^. Thus, nutrient deprivation determined various cancer cell biology. However the role of nutrient deprivation in cancer cell ferroptosis sensitivity has rarely been reported. According to the previous report that nutrient deprived cancer cells rely on endogenous fatty acid synthesis to maintain survival, we wondered the exact role of nutrient deprivation in cancer cell ferroptosis and the underlying mechanisms.

Here, we demonstrate that nutrient deprived pancreatic cancer cells exhibit resistance to ferroptosis. Mechanistically, nutrient-deprived cancer cell could activate the mTOR-SREBP1-SCD1 axis, which further upregulated intracellular MUFA content and ultimately lead to ferroptosis resistance. Moreover, rapamycin, an mTOR-specific inhibitor, reduced intracellular MUFAs and exhibited a synergistic therapeutic effect with ferroptosis inducers *in vitro* and *in vivo*. These findings establish a potential ferroptosis-mediated strategy for pancreatic cancer therapy.

## Results

### Nutrient-deprived facilitated pancreatic cancer cells ferroptosis resistance

**Figure 1:**
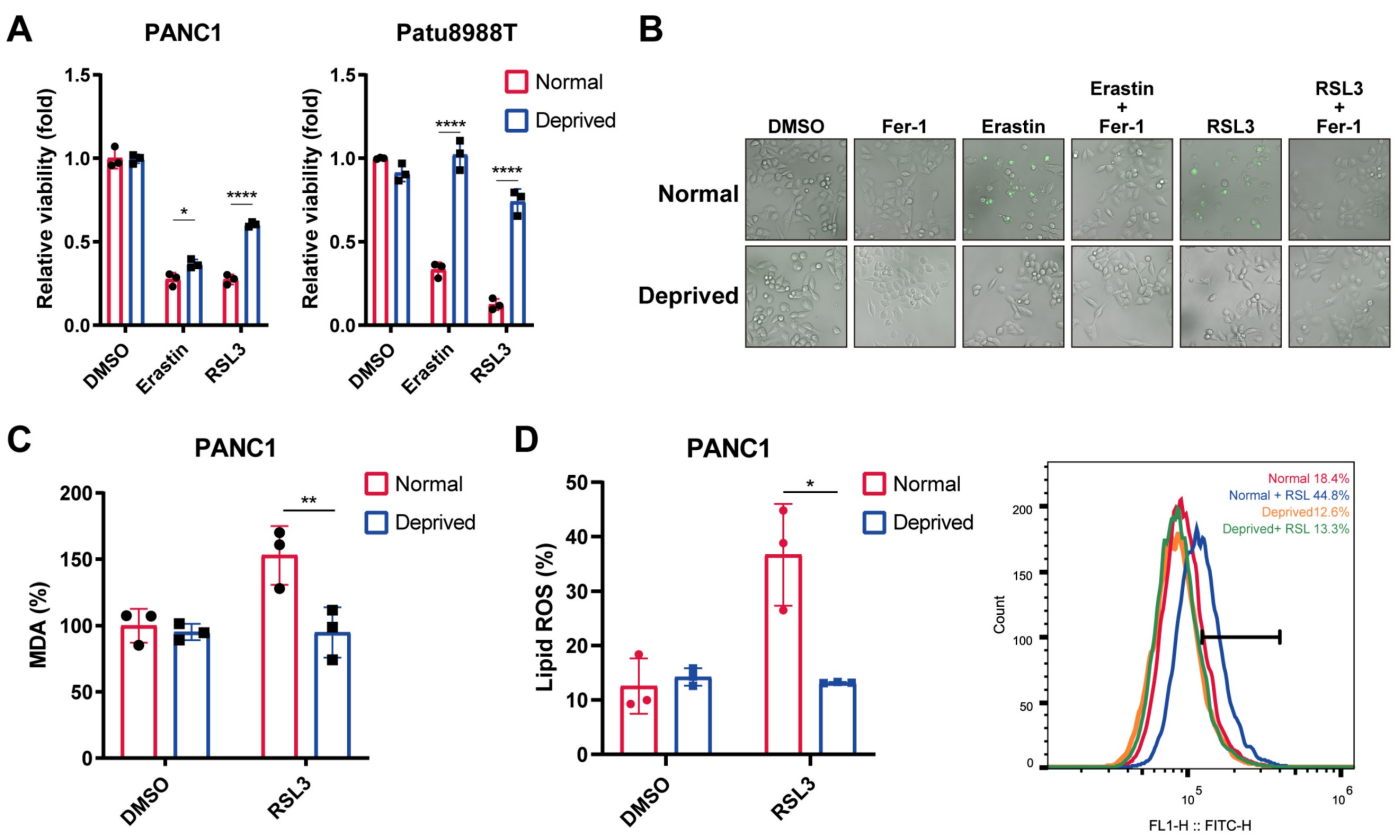
**Nutrient deprivation protects pancreatic cancer cells from ferroptosis. (A)** Relative viability of PANC1 and Patu8988T cells cultured under normal and nutrient-deprived medium treated with indicated ferroptosis inducers for 24 hours. **(B)** SYTOX green staining of PANC1 cells cultured under normal and nutrient-deprived medium treated with ferroptosis inducers or salvager for 24 hours. **(C)** Relative MDA levels of PANC1 cells cultured under normal and nutrient-deprived medium treated with RSL3 for 24 hours. **(D)** Lipid ROS levels of PANC1 cells cultured under normal and nutrient-deprived medium treated with RSL3 for 24 hours. Error bars represent SEM from three independent experiments. *p < 0.05, **p < 0.01, ***p < 0.001, ****p < 0.0001.

To investigate the exact role of nutrient-deprivation on the cancer cell ferroptosis, human pancreatic cancer cell lines (PANC1 and Patu8988T) were cultured in Dulbecco's Modified Eagle Medium (DMEM) supplemented with 2% fetal bovine serum (FBS), which mimicked the nutrient-deprivation condition according to the previous publication^21^. CCK8 assay and SYTOX staining revealed that nutrient deprivation markedly reversed erastin- or RSL3- induced cell death, resembling the effect exerted by ferrostatin-1 (Fer-1), a ferroptotic cell death inhibitor (Figure 1A, B).We next evaluated the alterations in two ferroptosis indicators, lipid ROS and malondialdehyde (MDA), an end product of lipid peroxidation. Interestingly, the levels of MDA and lipid ROS were significantly increased by RSL3, which could be reversed under nutrient-deprived condition (Figure 1C, D). Together, our results indicate that nutrient deprivation contributed to the pancreatic cancer cell ferroptosis resistance.

### Nutrient-deprived pancreatic cancer cells exhibit increased MUFAs biosynthesis mediated by SCD1

**Figure 2:**
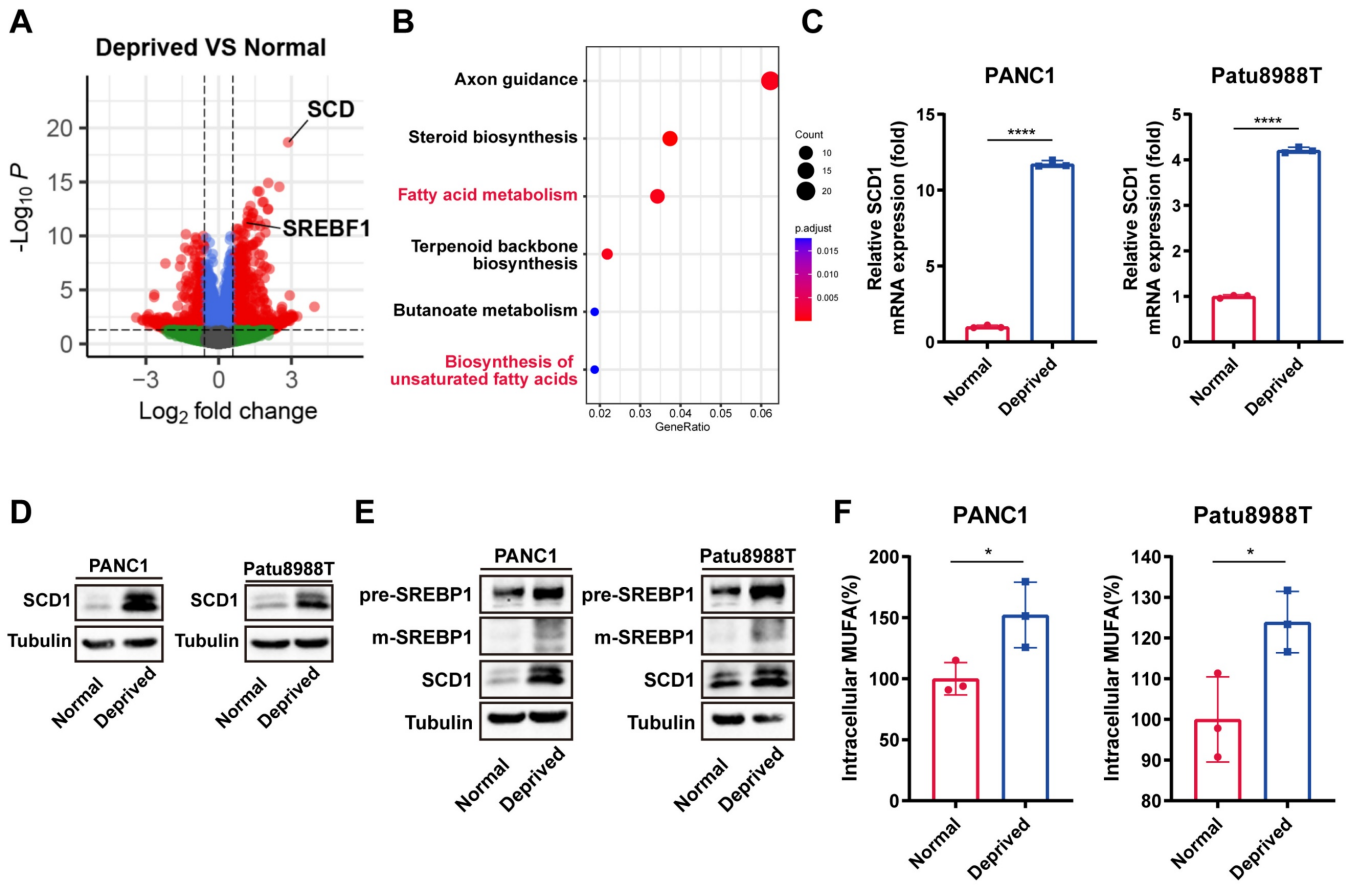
**SCD1 mediated MUFAs biosynthesis results in nutrient-deprived cells ferroptosis resistance**. **(A)** Volcano plot of differential gene expression analysis for nutrient-deprived and normal PANC1 cells. **(B)** KEGG enrichment analysis of upregulated expression genes using clusterProfiler R package. **(C)** RT-qPCR analysis of mRNA expression of SCD1 for nutrient-deprived and normal PANC1 and Patu8988T cells. **(D)** Immunoblot analysis of protein expression levels of SCD1 for nutrient-deprived and normal PANC1 and Patu8988T cells. **(E)** Immunoblot analysis of protein expression levels of precursor SREBP1, mature SREBP1 and SCD1 expression for nutrient-deprived and normal PANC1 and Patu8988T cells. **(F)** ELISA assay of relative intracellular MUFAs for nutrient-deprived and normal PANC1 and Patu8988T cells. Error bars represent SEM from three independent experiments. *p < 0.05, **p < 0.01, ***p < 0.001, ****p < 0.0001.

To further explore the underlying mechanisms of nutrient deprivation derived ferroptosis resistance, RNA-seq was performed in PANC1 cells cultured under normal and nutrient-deprived conditions. Differential gene expression analysis results showed that Stearoyl-CoA Desaturase-1 (SCD1) ranked first as the most significantly upregulated gene under nutrient-deprived conditions (Figure 2A). The KEGG pathway enrichment analysis indicated a notable upregulation of fatty acid biosynthesis pathways, particularly those associated with unsaturated fatty acid synthesis (Figure 2B). Accordingly, compared to the control group, SCD1 expression was upregulated at the mRNA and protein level under nutrient-deprived conditions (Figure 2C, D).

As the SREBP1-SCD1 axis functioned as the core pathway for de novo lipid synthesis within tumor cells, we wonder whether the SREBP1-SCD1 pathway involved in nutrient-deprived regulated ferroptosis resistance^22^. Immunoblot analysis showed the upregulation of precursor SREBP1 and mature SREBP1 at the protein level under the nutrient deprivation condition (Figure 2E). As the rate-limiting enzyme responsible for synthesis of monounsaturated fatty acids (MUFAs), the upregulation of SCD1 truly resulted in elevated levels of intracellular MUFAs according to ELISA assay under nutrient deprivation (Figure 2F). Together, these results indicated that cancer cells under nutrient deprivation activated the intrinsic SREBP1-SCD1 axis, which further synthesized more MUFAs to enhance the ferroptosis resistance ability.

### SREBP1-SCD1 axis contributed to nutrient-deprived pancreatic cancer cells ferroptosis resistance

**Figure 3:**
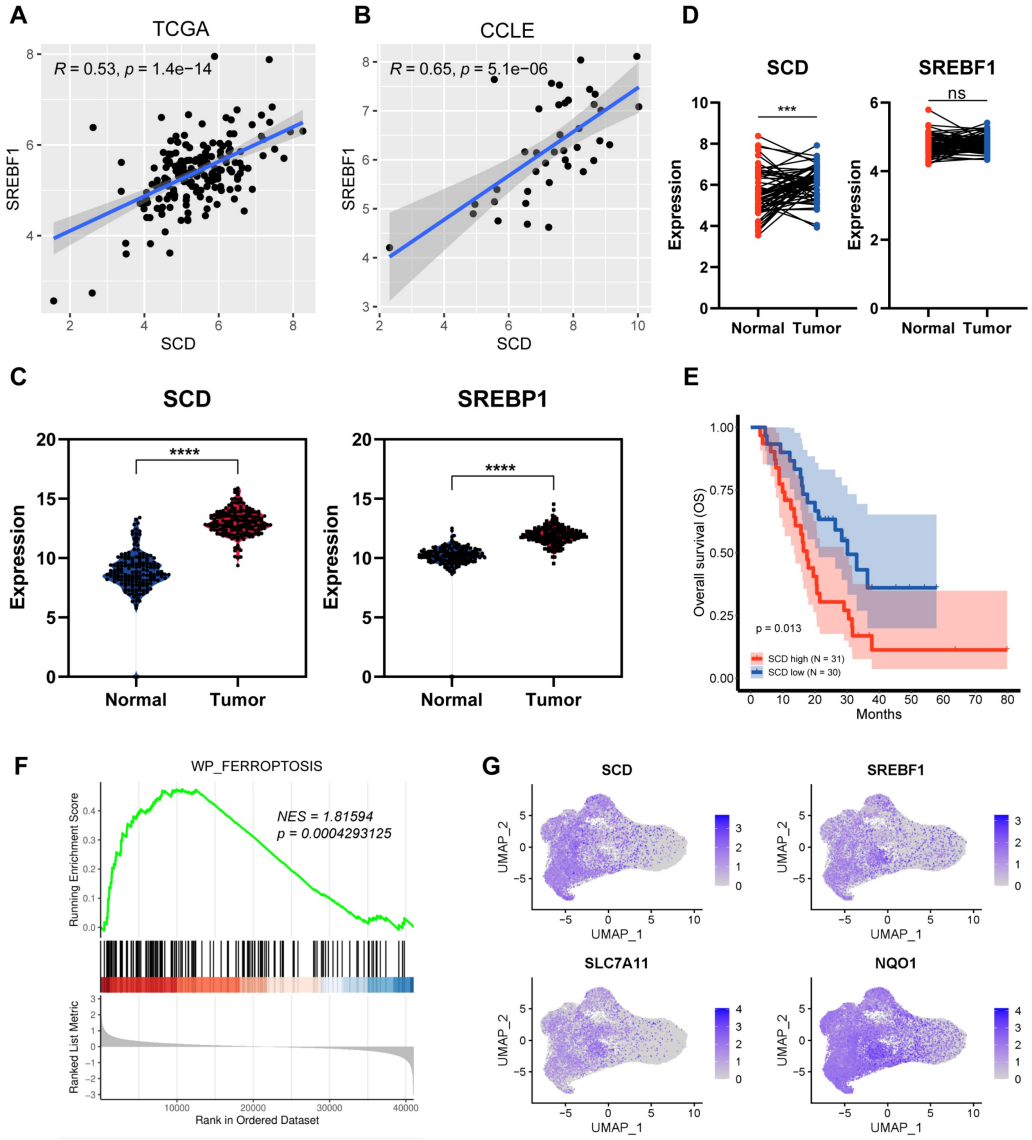
** SREBP1-SCD1 axis is closely associated with ferroptosis and tumorigenesis. (A), (B)** Correlation analysis of SREBP1 and SCD1 mRNA expression in TCGA database and CCLE database. **(C)** TCGA analysis of SCD1 and SREBP1 mRNA expression in pancreatic adenocarcinoma compared to normal pancreas. **(D)** Relative expression of 60 paired pancreatic cancer tissues with adjacent non-tumor tissues. **(E)** Kaplan-Meier curves of pancreatic cancer patients from GSE57495 database stratified by SCD1 mRNA expression (low vs high). **(F)** Gene Set Enrichment Analysis (GSEA) of differential genes compared SCD1 high group to low group. **G** Scatter plot of SCD1, SREBP1, SLC7A11 and NQO1 expression at single cell database.

As the critical regulator of fatty acid synthesis, we firstly investigate the clinical significance of SREBP1-SCD1 axis. Through the analysis of TCGA and CCLE databases, we found a positive and coordinated regulatory correlation between SREBP1 and SCD1 in tissue samples and cell lines (Figure 3A, B). From the pancreatic cancer patients TCGA database, SREBP1 and SCD1 expressed higher at the mRNA level in tumor tissues (Figure 3C). We also compared 60 paired pancreatic cancer tissues with adjacent non-tumor tissues and found that the expression level of SCD1 was higher in tumor tissues (Figure 3D). Kaplan-Meier analysis of the TCGA data, with patients divided into high- or low- SCD1 groups, showed a trend of higher overall survival rate associated with low SCD1 expression (Figure 3E). These results indicate that while the SREBP1-SCD1 axis is broadly upregulated in cancer, its ferroptosis-protective role appears to be particularly relevant in pancreatic cancer, where nutrient deprivation is a prominent microenvironmental stressor. The elevated expression of this axis in pancreatic tumors suggests a cancer-type-specific adaptation that enhances resistance to ferroptosis-induced cell death. Together, these findings suggest that SREBP1 and SCD1 are not only involved in lipid metabolism and tumorigenesis but also play a context-dependent role in ferroptosis resistance under nutrient-deprived conditions in pancreatic cancer.

To further assess the relationship between SCD1 and ferroptosis, we divided 61 pancreatic cancer samples into high and low SCD1 groups and further performed Gene Set Enrichment Analysis (GSEA). The GSEA analysis showed that ferroptosis pathway was significantly enriched in the SCD1 high groups (Figure 3F). Furthermore, we analyzed integrated single-cell atlas comprising ductal cell data from over 70 samples across six datasets^23^. We found that the expression of SREBP1 and SCD1 highly overlapped with common ferroptosis resistance molecules such as SLC7A11 and NQO1 (Figure 3G). These results suggest that SREBP1 and SCD1 are potential candidates involved in ferroptosis resistance and tumorigenesis.

**Figure 4:**
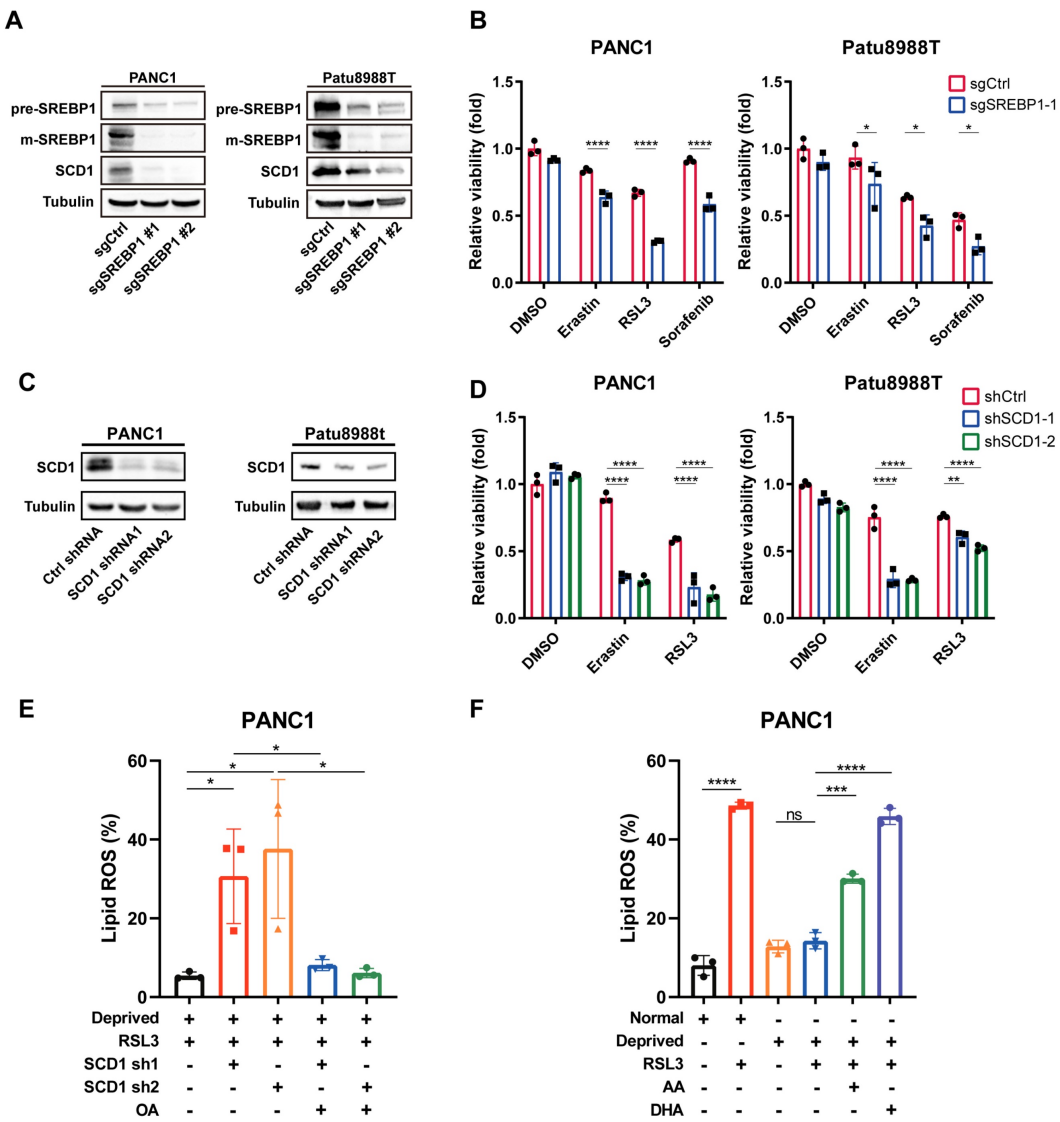
** SREBP1-SCD1 axis contributed to ferroptosis resistance in nutrient-deprived pancreatic cancer cells. (A)** Immunoblot analysis of protein expression levels of SREBP1 and SCD1 in negative control and SREBP1 knockout cells. **(B)** Relative cell viability of negative control and SREBP1 knockout cells treated with erastin, RSL3 or sorafenib (Sor) under nutrient deprivation for 24 hours. **(C)** Immunoblot analysis of SCD1 protein expression in control shRNA and SCD1 knockdown cell. **(D)** Relative cell viability of control shRNA and SCD1 knockdown cells treated with indicated ferroptosis inducers under nutrient deprivation for 24 hours. **(E)** Lipid ROS levels of control shRNA and SCD1 knockdown cells treated with RSL3 or OA (50 μM) under nutrient deprivation for 24 hours were detected using flow cytometry. **(F)** Lipid ROS levels of PANC1 cells cultured under normal and nutrient-deprived medium treated with RSL3, AA (20 μM) or DHA (20 μM) for 24 hours. Experiments were repeated at least three times, and the data are expressed as the mean ± SEM. *p < 0.05, **p < 0.01, ***p < 0.001, ****p < 0.0001.

To uncover whether SREBP1-SCD1 axis determined nutrient-deprived pancreatic cancer cells ferroptosis resistance, we employed CRISPR-Cas9 technology to knockout SREBP1 in pancreatic cancer cells with high efficiency (Figure 4A). Immunoblot analysis revealed that SCD1 expression level was downregulated in SREBP1-knockout cancer cell (Figure 4A). Interestingly, nutrient deprivation regulated ferroptosis resistance was abolished in SREBP1 knockout cancer cells (Figure 4B). Next, we performed shRNA to knockdown SCD1 in cancer cells (Figure 4C). Accordingly, SCD1 knockdown cancer cell exhibited increased cell death in the presence of ferroptosis inducers under nutrient deprivation (Figure 4D). In keeping with this phenotype, lipid reactive oxygen species (ROS) rose significantly in nutrient-deprived SCD1-knockdown cells relative to parental controls (Figure 4E). Remarkably, a pre-treatment with the MUFA oleic acid, which increases the cellular MUFA/PUFA ratio, completely rescued the lipid ROS surge (Figure 4E). Conversely, lowering the MUFA/PUFA ratio by enriching nutrient-deprived parental cells with the PUFAs arachidonic acid (AA) or docosahexaenoic acid (DHA) reinstated robust lipid peroxidation and eliminated the nutrient-deprivation-induced ferroptosis resistance (Figure 4F). Together, these findings demonstrate that SCD1-driven MUFA synthesis protects pancreatic cancer cells from ferroptosis by sustaining a high MUFA/PUFA balance, and they establish a direct causal link between this ratio and lipid peroxidation under metabolic stress.

### Rapamycin synergistic ferroptosis inducer via suppressing mTOR-SREBP1-SCD1 axis

**Figure 5:**
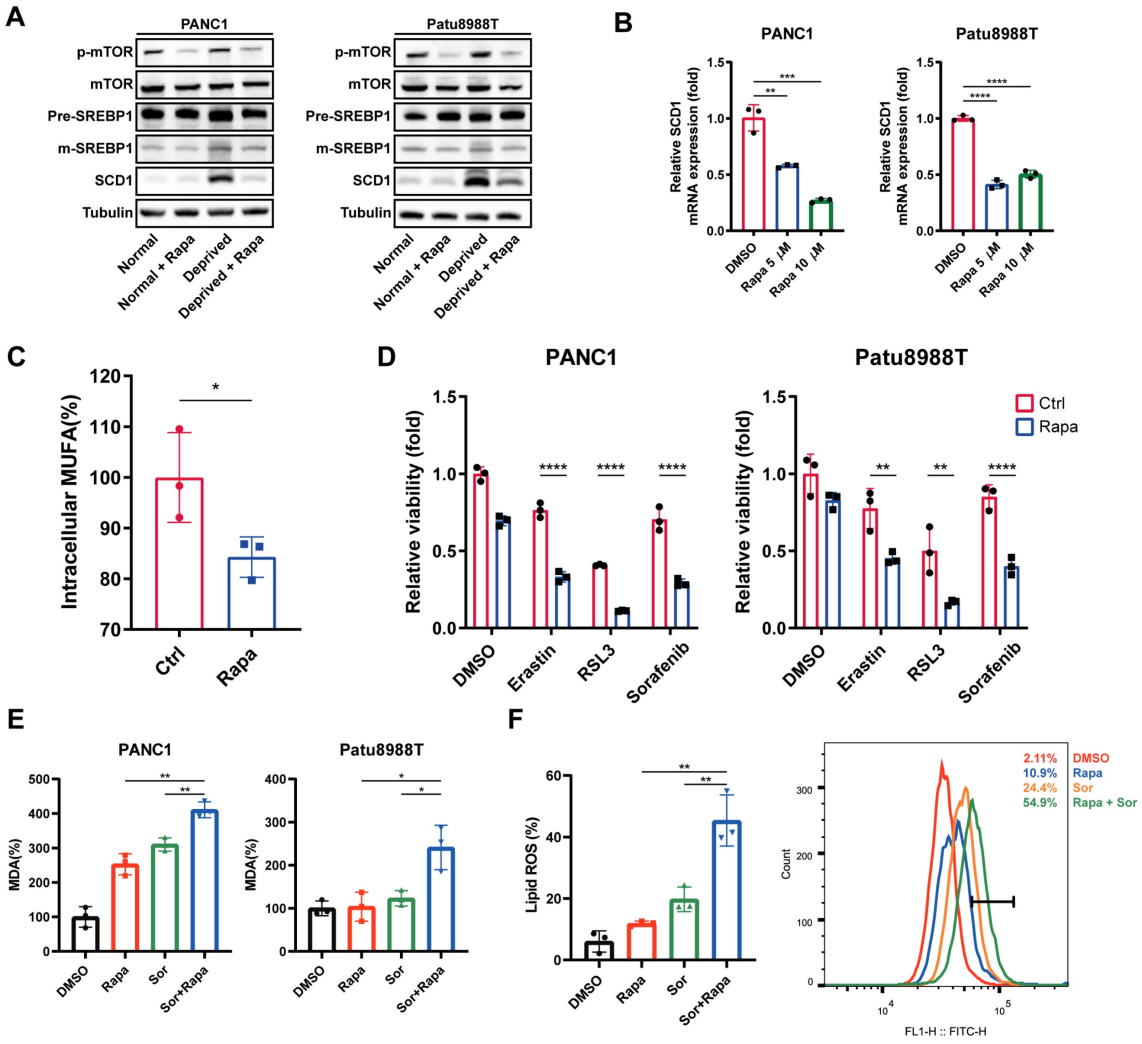
** Rapamycin sensitized nutrient-deprived pancreatic cancer cells to ferroptosis. (A)** Immunoblot analysis of p-mTOR, mTOR, SREBP1 and SCD1 protein expression treated with rapamycin (Rapa) for 24 hours under indicated conditions. **(B)** RT-qPCR analysis of SCD1 mRNA expression in nutrient-deprived pancreatic cancer cells treated with rapamycin (Rapa).** (C)** ELISA assay was performed to detect intracellular MUFAs content of PANC1 cells treated with rapamycin (Rapa) for 24 hours under nutrient deprivation. **(D)** Relative cell viability of nutrient-deprived cells treated as indicated. **(E)** Relative MDA levels of nutrient-deprived cells treated with rapamycin (Rapa) and sorafenib (Sor) for 24 hours. **(F)** Lipid ROS level of nutrient-deprived cells treated with rapamycin (Rapa) and sorafenib (Sor) for 24 hours. Experiments were repeated three times, and the data are expressed as the mean ± SEM. *p < 0.05, **p < 0.01, ***p < 0.001, ****p < 0.0001.

Given that the mTOR-SREBP1-SCD1 pathway is a classic lipid metabolism regulatory pathway, and the activation of the SREBP1-SCD1 axis was the underlying mechanism for nutrient deprivation-induced ferroptosis resistance, we will next explore the synergistic effect of mTOR complex 1 (mTORC1) inhibition on ferroptosis inducers. Rapamycin, a Food and Drug Administration (FDA)-approved drug for autoimmune diseases, was chosen as the mTOR inhibitor for *in vitro* and *in vivo* study. As expected, rapamycin (Rapa) strongly downregulated phosphorylated mTOR, mature SREBP, and SCD1 expression at the protein level under nutrient deprivation (Figure 5A). Also, RT-qPCR results showed that rapamycin significantly decreased mRNA levels of SCD1 (Figure 5B). Additionally, lower intracellular MUFAs concentration was acquired in nutrient-deprived cancer cells treated with rapamycin (Figure 5C).

Next, we investigated the synergistic effect of rapamycin with ferroptosis inducers under nutrient deprivation. Ferroptosis inducers, erastin, RSL3, and sorafenib (Sor), induced nutrient deprivation cancer cell death was further significantly enhanced in the presence of rapamycin (Figure 5D). Accordingly, compared to the control group (DMSO), rapamycin, or sorafenib treatment group, the MDA and lipid ROS levels in the rapamycin and sorafenib combination therapy group was significantly enhanced under nutrient deprivation (Figure 5E, F). These findings indicated that rapamycin sensitizes nutrient-deprived pancreatic cancer cells to ferroptosis, which contributed to reverse the nutrient deprivation induced ferroptosis resistance.

### Sorafenib and rapamycin combination therapy reverse nutrient deprivation cancer cell ferroptosis resistance *in vivo*

**Figure 6:**
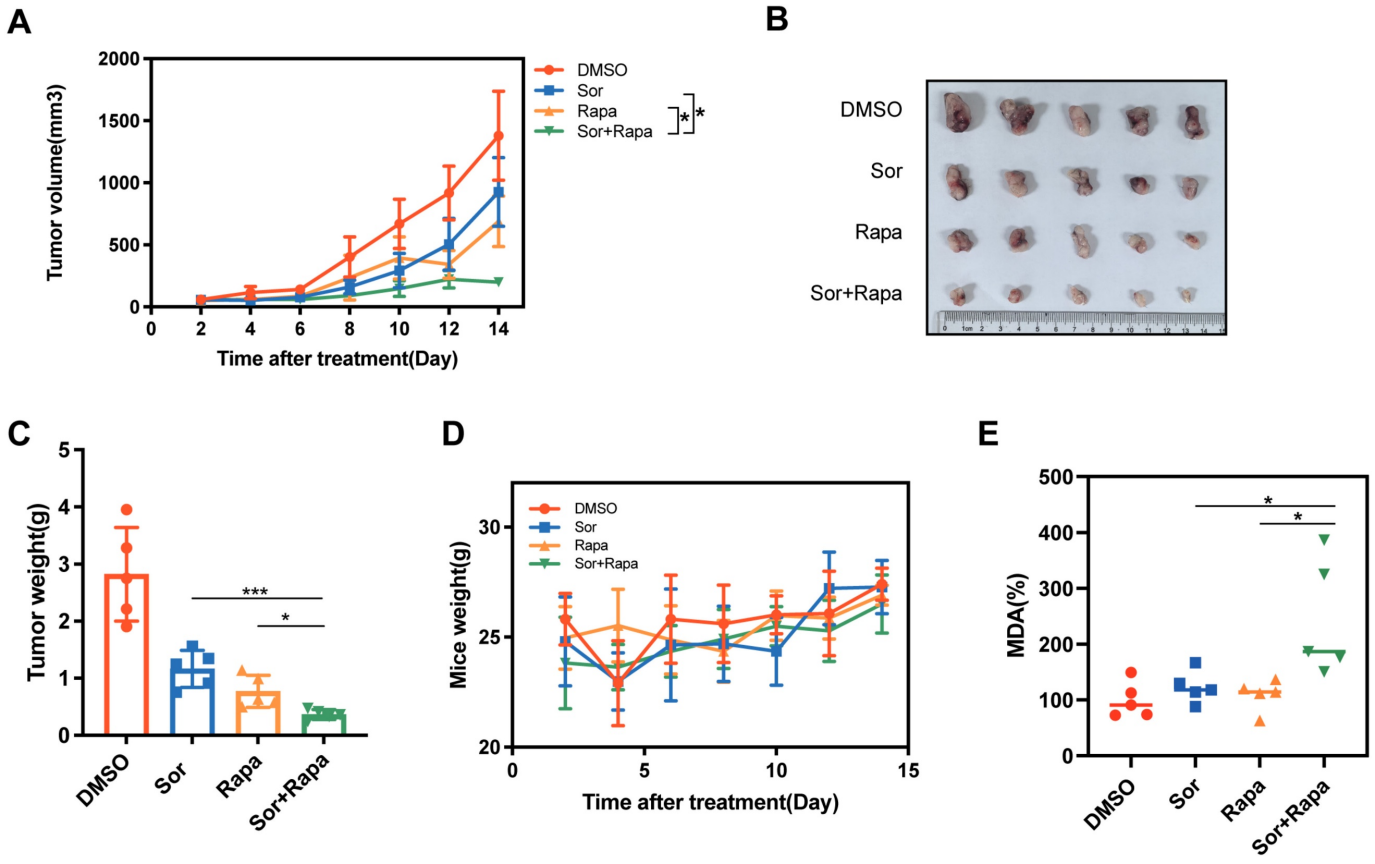
** Rapamycin enhanced sorafenib induced ferroptosis *in vivo*. (A)** Tumor growth curves for tumors treated with DMSO, sorafenib (Sor), rapamycin (Rapa) or sorafenib + rapamycin (Sor+Rapa). **(B)** Representative photographs of isolated tumor tissues following indicated treatments. **(C)** Tumor weight of isolated tumor tissues following indicated treatments. **(D)** Mice weight of each group. **(E)** Relative MDA levels of isolated tumor tissues in each group. *p < 0.05, **p < 0.01, ***p < 0.001, ****p < 0.0001.

To test whether the *in vitro* observation of nutrient deprivation regulated cancer cell ferroptotic cell death resistance could be replicated *in vivo*, C57BL/6 mice bearing Panc02 subcutaneous tumor were treated with the following groups, (1) DMSO (control group), (2) sorafenib (Sor), a clinically used chemotherapy drug and ferroptosis inducer, (3) rapamycin (Rapa), (4) sorafenib and rapamycin combination (Sor+Rapa). Combined sorafenib and rapamycin treatment achieved greater suppression of tumor volume and tumor weight than the other three groups (Figure 6A-C). The combination treatment displayed the least body weight, which may be ascribed to the reduced tumor weight (Figure 6D). Furthermore, the combination treatment showed elevated levels of MDA, indicating increased lipid peroxidation and ferroptosis in tumor tissues (Figure 6E). Collectively, these results suggested that combination of sorafenib and rapamycin exhibited a great synergistic antitumor effect through utilizing ferroptosis *in vivo*.

## Discussion

**Figure 7:**
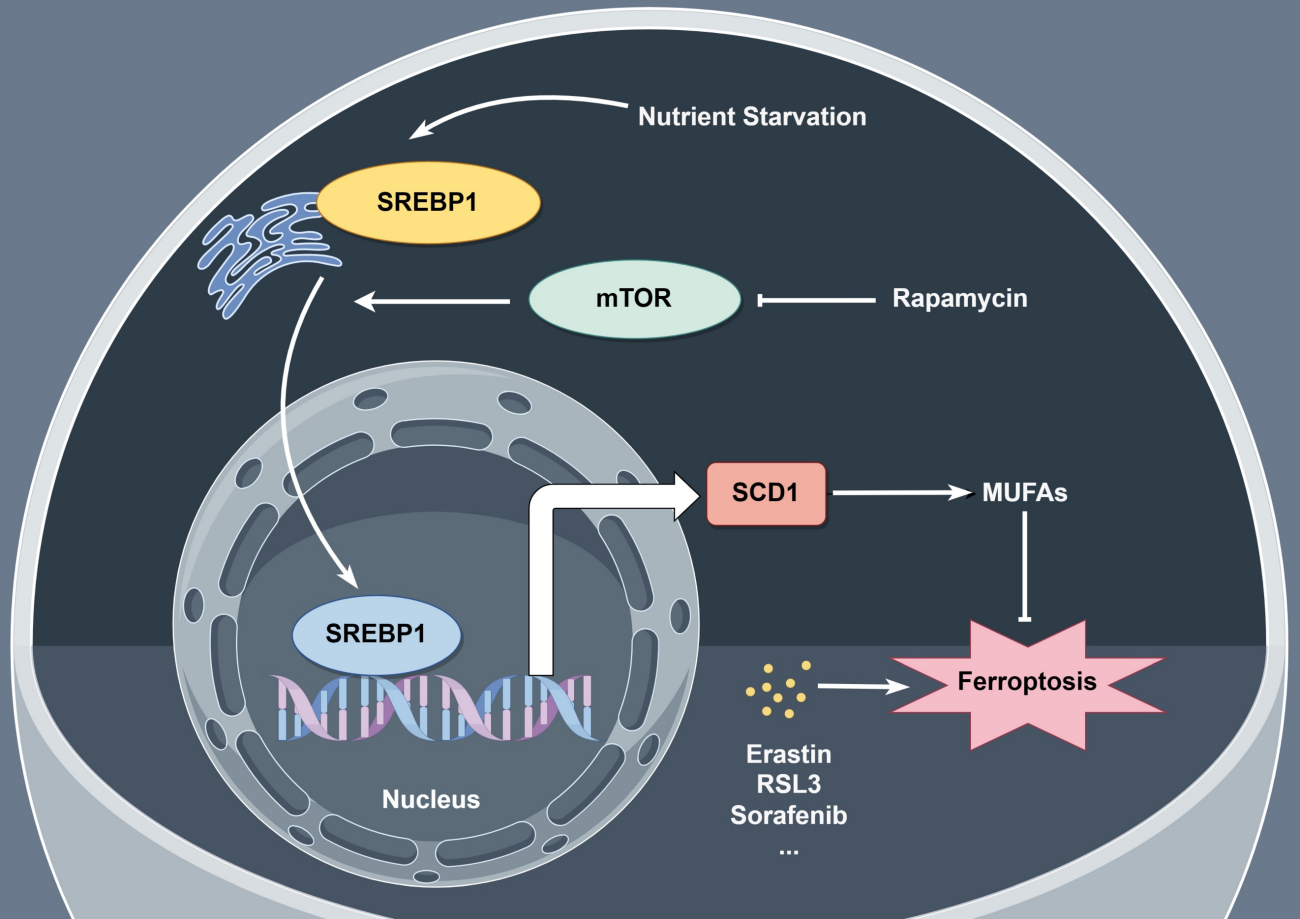
** Schematic representation of mechanism of ferroptosis resistance in pancreatic cancer cells under nutrient deprivation.** Nutrient-deprived microenvironment activates the mTOR-SREBP1-SCD1 axis in pancreatic cancer cells, promoting the biosynthesis of MUFAs within the cells, leading to resistance to ferroptosis. The mTOR inhibitor rapamycin suppresses SCD1 expression, thereby inhibiting the biosynthesis of MUFAs within the cells and sensitizing nutrient-deprived pancreatic cancer cells to ferroptosis.

Despite the progress of modern medicine, pancreatic cancer is the fourth leading cause of death in developed country. PDAC is notoriously resistant to current therapies due to its complicated tumor environment featured with lacking in nutrients and oxygen. Here, we aimed to figure out the mechanism of PDAC cells ferroptosis resistance under nutrient deprived condition. Activation of SREBP1-SCD1 axis facilitates the intracellular MUFA biosynthesis which may increase the ratio of MUFAs to PUFAs and result in ferroptosis resistance. Additionally, rapamycin, a specific mTOR inhibitor, shows a promising tumor inhibitory effect combining with ferroptosis inducers in *vitro* and *in vivo* (Figure 7).

In PDAC, nutrient deprived microenvironment plays a vital role in tumor growth and malignancy. PDAC cells have to develop a series of metabolic reprogramming mechanisms to meet the metabolic needs for tumor progression. However, the exact mechanisms for this process are poorly characterized. To mimic such nutrient deprived condition, we appropriately reduced the content of serum in culture medium, which contains diverse nutrient. Through whole-genome transcriptome sequencing, we found that PDAC cells upregulated their intracellular lipid biosynthesis pathways for cells survival, among which SREBP1-SCD1 mediated MUFAs production occupies a major position.

By catalyzing the conversion of saturated fatty acids (SFA) to monounsaturated fatty acids (MUFA), SCD1 maintains a crucial balance between SFA and MUFA, which is essential for regulating lipid biosynthesis and cellular metabolism. Increasing evidence suggests that SCD1 plays a crucial role as a regulator of ferroptosis. Ferroptosis is a form of iron-dependent, non-apoptotic cell death mechanism initiated by the accumulation of oxidized PUFAs, which are subsequently incorporated into the phospholipid cell membrane. Compared to PUFAs, MUFAs are less susceptible to oxidation. Therefore, in cancer cells, the increased production of MUFAs induced by SCD1 overexpression can prevent ferroptosis. Our study is the first to identify a significant upregulation of SCD1 under nutrient-deprived conditions, leading to enhanced generation of MUFAs in cancer cells. This may explain, at least in part, the resistance of pancreatic cancer cells to various ferroptosis treatment modalities, such as radiotherapy, is due to nutrient-deprived microenvironment.

Rapamycin is a widely used immuno-suppressant and antitumor drug in the biomedical field, sparking broad research interest in cellular biology and molecular medicine. Recent studies have found that rapamycin exhibits promising anti-aging effects and inhibits tumor cell proliferation^24^,^25^. The results of this study further confirm the inhibitory effect of rapamycin on MUFAs synthesis in nutrient-deprived pancreatic cancer cells, supporting its role as a synergistic drug in ferroptosis treatment strategies. Although the combination of rapamycin and sorafenib demonstrates promising tumor-suppressive effects in this study, further exploration of additional ferroptosis induction modalities and the development of more effective administration strategies are needed to enhance their efficacy and safety in cancer therapy.

Altogether, we show that SREBP1-SCD1 dependent lipid synthesis under nutrient deprivation drives ferroptosis resistance in pancreatic cancer. Rapamycin holds promise as a novel ferroptosis sensitizer.

## Materials and Methods

### Cell culture and agents

Human pancreatic cancer cell lines (PANC1 and Patu8988T) and mice pancreatic cancer cell line Panc02 were obtained from Procell Life Science & Technology Company (China). Pancreatic cancer cells were cultured in high glucose Dulbecco's modified Eagle medium (DMEM) supplemented with 10% fetal bovine serum and antibiotics (100 units/mL penicillin, 100 mg/mL streptomycin). Nutrient-deprived condition was performed in DMEM with 2% FBS and antibiotics (100 units/mL penicillin, 100 mg/mL streptomycin). They were maintained at 37°C in 5% CO2, with saturated humidity.

Erastin (HY-15763), RSL3 (HY-100218A), Sorafenib (HY-10201), Rapamycin (HY-10219), ferrostatin-1 (HY-100579), Z-VAD-FMK (HY-16658) and Necrosulfonamide (HY-100573) were purchased from MedChemExpress (MCE, USA). Oleic acid (T2O2668), arachidonic acid (T4129) and docosahexaenoic acid (T5369) were obtained from TargetMol.

### Cell viability assay

Cells were seeded into 96-well plates and incubated with the indicated treatments. Cell viability was typically assessed by Cell Counting Kit-8 (APExBIO, K1018). Briefly, 100μl fresh medium was added to cells containing 10μl CCK8 solutions and incubated for 1-4h (37°C, 5% CO2). Absorbance at wavelengths of 450 nm was measured using a microplate reader. Cell viability was calculated as a percentage relative to the negative control. SYTOX green staining (S7020, Thermo) was used to detect cell death.

### Lipid peroxidation assay

The relative MDA level was measured using a Lipid Peroxidation (MDA) Assay Kit Nanjing Jiancheng (#A003-1-2) according to the manufacturer's instructions. Cells or tumor tissues were homogenized with lysis buffer and the MDA in samples reacts with thiobarbituric acid (TBA) to generate an MDA-TBA adduct which can be quantified colorimetrically (OD 532 nm).

### Flow Cytometry

To access reactive oxygen species in membrane lipids via a shift of fluorescence emission peak upon oxidation of its polyunsaturated butadienyl group, cells were treated as indicated, then harvested and incubated with C11 BODIPY (Abclonal) for 20 minutes at 37°C. Cells were washed and resuspended in 500 μL fresh PBS, then analyzed immediately with a flow cytometer.

### Western blot

Cells were collected and lysed with 1× cell lysis buffer (Leagene, PS0009) on ice for 30 min and centrifuged at 12,000×g for 5 min at 4°C. Protein was quantified using the BCA assay (Thermo Fisher Scientific, 23225). Western blotting assay was performed as described previously. Antibodies were mTOR (Proteintech, #66888-1-Ig, 1:10000), Phospho-mTOR (Ser2448) (Proteintech, #67778-1-Ig, 1:5000), SREBP1 (SantaCruz, #sc13551, #sc-13551), SCD1 (Abcam, #ab19862, 1:1000), and β-tubulin (Abcam, #ab6046, 1:1000). Secondary antibody (either anti-rabbit or anti-mouse) was purchased from Boster Biotechnology Company (China). The blots were analyzed using the software ImageJ (Version 1.80, NIH, USA).

### Quantitative real-time PCR

Total RNA was extracted using RNAiso Plus (Takara) according to the manufacturer's instructions. For mRNA analysis, RevertAid First-Strand cDNA Synthesis Kit (Thermo Fisher Scientific) was performed for reverse transcription according to the manufacturer's instructions. Subsequently, SYBR Green-based real-time PCR was performed in triplicate using SYBR Green master mix (Thermo Fisher Scientific) on a QuantStudio 3 real time PCR machine (Thermo Fisher Scientific). For analysis, the comparative CT values for each gene were normalized to those of ACTB. The following gene-specific primers were used:

SCD1-F: CCTGGTTTCACTTGGAGCTGTG; SCD1-R: TGTGGTGAAGTTGATGTGCCAGC; ACTB-F: CACCATTGGCAATGAGCGGTTC; ACTB-R: AGGTCTTTGCGGATGTCCACGT.

### CRISPR-Cas9 assay

Non-targeting and SREBP1 knockout lentivirus were purchased from GENECHEM. PANC1 and Patu8988T SREBP1 knockout cell line was generated via lentivirus infection and puromycin selection according to the manufacturer's instructions. The following targeting sequences are used:

SREBP1-sgRNA1: CGGG-TACATCTTCAATGGAG; SREBP1-sgRNA2: TAGGGTGGGTCAAATAGGCC; Nontargeting sequence: CGCTTCCGCGGCCCGTTCAA.

### RNAi and lentivirus infection

To generate SCD1 knockdown cells, cells were infected with lentivirus carrying SCD1-shRNA followed by puromycin (2 μg/ml) selection for 10-14 days. These established stable cell lines were maintained in DMEM containing 10% FBS and puromycin (0.75 μg/ml) for further experiments. The following specific shRNA sequences are used: SCD1-shRNA1: CCGGCGTCCTTATGACAAGAACATTCTCGAGAATGTTCTTGTCATAAGGACGTTTTTG; SCD1-shRNA2: CCGGCTACGGCTCTTTCTGATCATTCTCGA-GAATGATCAGAAAGAGCCGTAGTTTTTG.

### RNA sequencing

Total RNA was extracted from cells of the control and treatment groups using Trizol. After integrity and concentration assessment, 2 μg of RNA were used for sequencing library preparation. After library quality control, the indexed samples were clustered and sequenced on an Illumina platform, generating 150 bp paired-end reads. After trimmed, clean reads were mapped to the human genome reference (GRCh38 release109) retrieved from the Ensembl database (http://www.ensembl.org) using HISAT2_v2.2.1 software. The reads were then quantified with featureCounts_v2.0.1. Differential gene expression analysis was conducted using the limma_voom pipeline (limma_v3.54.0), with results presented in Supplementary Table 1.

### Xenograft tumor models

Five-week-old female C57BL/6 mice were obtained from GemPharmatech Company and maintained in Animal Center of Jiangsu University in compliance with the Guideline for the Care and Use of Laboratory Animals (NIH Publication No. 85-23, revised 1996). Panc02 cells (1×10^6^) were injected subcutaneously into the right dorsal flanks of C57BL/6 mice. The mice were randomized into four groups when tumors reached a volume of 50-100mm^3^ and treated with DMSO (control), sorafenib (10 mg/kg), rapamycin (3 mg/kg), or combination of two drugs by intraperitoneal injection every two days for two weeks. The tumor volume and growth speed were monitored every two days until the end point at day 14. Animal studies were approved by the Committee on the Use of Live Animals for Teaching and Research of Jiangsu University.

### Public dataset analysis

To determine the expression of SCD1 and SREBP1 in pancreatic cancer and normal pancreas tissues, the datasets in The Cancer Genome Atlas (TCGA) and Genotype-tissue expression project (GTEx) were adopted (https://xenabrowser.net/datapages/). To compared 60 paired pancreatic cancer tissues with adjacent non-tumor tissues, gene expression data was downloaded from Gene Expression Omnibus (GEO) database (GSE62452). To analyze the prognosis and GSEA enrichment pathway of SCD1 high and low patients, gene expression and patients' survival data were obtained from Gene Expression Omnibus (GEO) database (GSE57495). For analysis of gene expression at single cell level, integrated single-cell data from over 70 samples were utilized (zenodo [10.5281/zenodo.6024273]).

### Statistical analysis

All data are presented as the mean ± standard error of the mean (SEM). Statistical analysis was performed using Prism 8 software. Differences between means were determined using Student's t tests, one-way analysis of variance (ANOVA) or two-way ANOVA, and were considered significant at p < 0.05. All the experiments were repeated at least three times.

## Supplementary Material

Supplementary table.
